# Harm reduction self-efficacy and motivations for contactless supply access among a sample of syringe services program participants

**DOI:** 10.1186/s12954-025-01288-8

**Published:** 2025-07-28

**Authors:** Rachel A. Hoopsick, Benjamin M. Campbell, R. Andrew Yockey

**Affiliations:** 1 Department of Health and Kinesiology, University of Illinois Urbana-Champaign, 1206 S. Fourth St., 2017 Khan Annex, Huff Hall, Champaign, IL 61820 USA; 2 Department of Public Health, University of Mississippi, Oxford, MS 38677 USA

**Keywords:** Harm reduction access, Syringe service access, Harm reduction vending machines, Barriers to harm reduction

## Abstract

**Background:**

Contactless harm reduction supply methods (e.g., vending machines, mail order, mobile delivery) have become prevalent in the United States. However, this approach has faced some criticisms, including the notion that, unlike staffed syringe services programs, contactless methods do not provide face-to-face support, education, or referrals to treatment, potentially limiting their overall impact.

**Methods:**

We collected self-reported data from a sample of people who inject drugs who accessed a syringe services program (N = 50), including their demographics, harm reduction self-efficacy (i.e., confidence to employ specific health-preserving coping skills in high-risk drug using situations), and motivations for contactless harm reduction supply access via vending machine. We explored differences in the participants' demographics and harm reduction self-efficacy by usual method of harm reduction supply access (in-person vs. vending machine).

**Results:**

Participants accessed the harm reduction supply vending machine primarily out of convenience (66%) and limited syringe services program hours (56%). Fear of being seen by someone they knew (28%), law enforcement (34%), and social services (22%) were also motivators. Overall, harm reduction self-efficacy was highest for safer injection practices but lowest for reducing drug use. We did not find any significant differences in participants’ demographics or harm reduction self-efficacy by access method.

**Conclusions:**

People who access harm reduction supplies in person and through contactless methods may not meaningfully differ in terms of their demographics and harm reduction self-efficacy, and contactless harm reduction supply methods are more convenient than in-person services. Findings support continued reductions to barriers of harm reduction services.

## Introduction

Syringe services programs (SSPs; e.g., needle exchange programs [NEPs], needle and syringe programs [NSPs]) are pivotal to reducing the harms associated with injecting drugs in the context of the illicit unregulated drug market in North America [25], offering access to sterile syringes and related supplies, thereby reducing the risk of infectious diseases and promoting safer injection practices for people who inject drugs [16]. While many SSPs provide in-person services, the emergence of contactless methods, including delivery, vending machines, and other discreet pickup options, has expanded accessibility [9, 21]. Despite reducing barriers to accessing harm reduction supplies, contactless methods have faced challenges due to historical stigmatization and political resistance [13, 23], including concerns about public visibility, perceived facilitation of drug use, and lack of oversight [22]. Most notably, a significant criticism of this approach has been that, unlike staffed SSPs, contactless methods do not provide face-to-face support, education, or referrals to treatment [2, 22].

Harm reduction vending machines have gained attention as a strategy to expand access to sterile syringes, naloxone, and other supplies, especially in settings where traditional services are limited [7, 23]; notably, Canada was an early adopter of this approach and serves as a successful model for implementation [4]. Recent studies highlight their potential to reach underserved populations [10, 27], while also raising important considerations around engagement, equity, and relational support [2, 22]. For example, Shaw and colleagues (2025) found notable differences between harm reduction vending machines and staffed services in providing education and referrals, and Bardwell and colleagues (2024) described how some people who use drugs viewed machines as more private and less stigmatizing. While much of the existing literature has focused on urban centers or large-scale program implementation, there remains a relative lack of research examining harm reduction services in mid-sized and smaller jurisdictions, which may face unique challenges related to infrastructure, stigma, and resource allocation.

People who use drugs often acquire harm reduction and drug use knowledge through informal social networks, particularly from more experienced peers [3, 14]. Increasingly, harm reduction information is also accessed through online sources, printed materials, and outreach from health organizations (e.g., National Harm Reduction Coalition, Canadian AIDS Treatment Information Exchange). These channels of information dissemination, alongside access to sterile supplies, may enhance individuals’ confidence in their ability to use drugs more safely. More research is needed to understand why people who inject drugs engage with contactless harm reduction services and how this access method might affect their knowledge of and ability to engage in harm-reducing behaviors. This study is informed by Bandura’s self-efficacy theory, which posits that confidence in one’s ability to perform specific behaviors influences action [1]. Contactless access methods like vending machines may enhance self-efficacy by reducing logistical and social barriers, while in-person SSPs may promote self-efficacy through direct education and supportive interactions with staff.

This brief report assesses the motivations of people who inject drugs to use a harm reduction vending machine and compares the demographics and harm reduction self-efficacy of individuals using in-person versus an unstaffed vending machine to access harm reduction supplies. By analyzing self-reported data from a pilot study of SSP participants from the state of Illinois (N = 50), this research aims to inform the improvement of harm reduction supply distribution strategies for people who inject drugs. Like most regions of the United States, the state of Illinois has seen a rise in drug poisoning fatalities in recent years, especially those involving opioids and psychostimulants [11].

## Methods

Participants for this pilot study were recruited from a small SSP located in central Illinois in a metro area with fewer than 250,000 residents [24], and this study was conducted as a part of a community-academic partnership in which SSP staff defined an area of needed research that would inform their practices. The SSP was embedded within a local health department that provides other onsite and mobile public health services. At the time of data collection, the SSP offered needs-based syringe distribution and disposal, naloxone provision and training, harm reduction education, infectious disease screening, and referrals to various co-located services, including dental care, family planning, immunizations, and food assistance. While most people served by the SSP lived in the largest cities within the same county as the local health department, some participants lived in more rural areas or other nearby Illinois counties, and some participants traveled from the state of Indiana, where many localities do not have SSPs.

The study included 50 adult participants who reported using injection drugs and had previously accessed harm reduction supplies at this SSP, recruited either in person or through flyers placed in a contactless vending machine outside the SSP facility. Both the contactless vending machine and in-person SSP services included the following supplies at the time of data collection: naloxone, syringes in various needle gauges, cookers, cotton, tourniquets, alcohol prep pads, bandages, sterile water, and sharps containers. At the time of this study, the health department building was open from 8:00 AM to 4:00 PM Monday through Friday for in-person services, but contactless supplies were available from a vending machine behind the building after hours and on weekends/holidays. Anyone was able to access the vending machine (e.g., no tokens, payment, identification, or other approval needed). After providing informed consent, eligible individuals completed a short online survey via Qualtrics (using either a tablet provided onsite by a member of the research team or their own internet-connected device), which took approximately 10 to 15 min. Participants were compensated with $10 in cash upon completion. The University of Illinois at Urbana-Champaign Institutional Review Board approved the study protocol. There were no instances of missing data across the items of this brief survey presented in the current study.

### Measures

Participants self-reported their demographics, including age, gender identity, race and ethnicity, educational attainment, employment status, annual household income, and living situation. Participants were also asked, “How do you usually access syringe services?” and they could indicate if they usually accessed harm reduction supplies in-person (walk-in or appointment inside the SSP building) or via a contactless vending machine outside of the SSP.

For this study, we also worked with SSP staff to develop a question about what might motivate participants to use a contactless method of accessing harm reduction supplies based on prior conversations that staff had with program participants. Participants were asked to “Check all of the following statements that apply to your experiences regarding in-person syringe services” with the following response options:

- “I can’t make it there during the times when it is open.”
- “I was afraid that someone I know would see me there”
- “I did not want to be lectured about my drug use”
- “I was afraid that the staff would judge me”
- “I was afraid that law enforcement would be there or find out that I had gone”
- “I was afraid that social services would be there or find out that I had gone”
- “The vending machine is more convenient than coming in for syringe services.” Participants also had the option to fill in a free-text box with additional comments. Responses were brief and varied in length, reflecting spontaneous comments rather than structured qualitative interviews or focus groups. Due to the brevity of responses and limited contextual detail, we chose to present all comments received to maintain transparency and avoid selective reporting. While this approach limited our ability to conduct a comprehensive thematic or demographic analysis, participant reflections nonetheless offer valuable insight into experiences and perceptions that complement the quantitative findings.

We also assessed participants' harm reduction self-efficacy (i.e., confidence to employ specific health-preserving coping skills in high-risk drug using situations) using a modified version of the Harm Reduction Self-Efficacy Questionnaire [20]. This measure assesses individuals’ harm reduction self-efficacy across 15 items on a Likert scale ranging from 0 (Not at all confident) to 10 (Very confident). Total Harm Reduction Self-Efficacy scores range from 0 to 150, with higher scores indicating great self-efficacy. This measure has been used in multiple studies with people who inject drugs and has demonstrated strong internal consistency and construct validity. The Harm Reduction Self-Efficacy Questionnaire also aligns with Bandura’s self-efficacy theory, which posits that individuals’ beliefs in their ability to perform specific behaviors are central to behavior change. The wording of several items was slightly adapted for our study based on the feedback of SSP staff to more closely align with the language used by SSP staff and SSP participants (e.g., replacing “dirty needle” with “used syringe”). The specific wording of individual items used in the current study is shown in Table 2. This measure had good internal consistency in our sample (α = 0.90).

### Analytic plan

We used descriptive statistics to characterize the sample and describe participants’ motivations for contactless supply access. We used bivariate analyses to examine for differences in the demographics and harm reduction self-efficacy of people who inject drugs according to whether they usually accessed the SSP in-person (via walk-in or appointment) or through a contactless supply vending machine located outside of the SSP building. All analyses were conducted with Stata/MP version 18.5 (College Station, TX) and were adjusted for multiple comparisons with a Bonferroni correction (23 comparisons, adjusted p < 0.0022).

## Results

### Demographics

Our sample of participants ranged in age from 23 to 65 years old and consisted of men (n = 22), women (n = 27), and nonbinary people (n = 1). The most commonly reported substances used by participants were illicit opioids (82.0%), followed by methamphetamine (58.0%) and cannabis (54.0%). Over one-third of participants reported using sedatives (36.0%) and cocaine (36.0%), while fewer reported using prescription opioids (28.0%) or other substances (20.0%). There were no statistically significant differences by usual harm reduction supply access method in participant age, gender identity, race/ethnicity, educational attainment, employment status, income, or living situation. Additional details are shown in Table 1.

**Table 1:** Demographics of people who inject drugs by usual harm reduction supply access method (N = 50)

	Overall sample (*N* = 50) mean (SD) or % (n)	In-person access (*n* = 12) mean (SD) or % (n)	Contactless access (*n* = 38) Mean (SD) or % (n)	p-value
Age, years	34.8 (8.6)	36.3 (8.0)	34.3 (8.8)	0.478
*Gender identity*				
Woman Man Nonbinary or genderqueer	54.0% (27) 44.0% (22) 2.0% (1)	50.0% (6) 50.0% (6) 0.0% (0)	42.1% (16) 55.3% (21) 2.6% (1)	0.782
*Race and ethnicity*				
Non-hispanic white Hispanic More than one race	90.0% (45) 8.0% (4) 2.0% (1)	91.7% (11) 8.3% (1) 0.0% (0)	89.5% (34) 7.9% (3) 2.6% (1)	0.851
*Education*				
Less than high school High school diploma or equivalent At least some college	16.0% (8) 54.0% (27) 30.0% (15)	16.7% (2) 58.3% (7) 25.0% (3)	15.8% (6) 52.6% (20) 31.6% (12)	0.935
*Employment status*				
Unemployed or disabled Working part-time Working full-time	52.0% (26) 32.0% (16) 16.0% (8)	41.7% (5) 25.0% (3) 33.3% (4)	55.3% (21) 34.2% (13) 10.5% (4)	0.239
*Income*				
Less than $10,000 $10,000–$29,999 $30,000–$49,999 $50,000 or more	40.0% (20) 38.0% (19) 16.0% (8) 6.0% (3)	33.3% (4) 33.3% (4) 33.3% (4) 0.0% (0)	42.1% (16) 39.5% (15) 10.5% (4) 7.9% (3)	0.130
*Living situation*				
In a house or apartment In my car, unsheltered on the street, under a bridge, etc Motel or hotel Other	72% (36) 16% (8) 6.0% (3) 6.0% (3)	75.0% (9) 16.7% (2) 0.0% (0) 8.3% (1)	71.1% (27) 15.8% (6) 7.9% (3) 5.3% (2)	0.392
*Drug use*				
Illicit opioids Methamphetamine Cannabis Sedatives Cocaine Prescription Opioids Other	82.0% (41) 58.0% (29) 54.0% (27) 36.0% (18) 36.0% (18) 28.0% (14) 20.0% (10)	– – – – – – –	– – – – – – –	–
*Usual harm reduction supply access method*				
In-person (walk-in or appointment) Contactless	24.0% (12) 76.0% (38)	– –	– –	–

### Motivations for contactless supply

A majority of participants (66.0%) reported accessing syringe services via contactless vending machines because they thought that this method was more convenient than coming in person. Similarly, more than half of participants (56.0%) reported accessing these services because they could not make it to the SSP when it was open. Some participants reported being afraid that someone they knew would see them inside the SSP building (28.0%) or that law enforcement (34.0%) or social services (22.0%) would be there or would find out that they had been there. A small proportion of participants reported using the contactless access method because they did not want to be lectured about their drug use (16.0%) or they were afraid that the SSP staff might judge them (18.0%).

Participants’ free-text comments provided in the survey highlighted both structural and geographic barriers to accessing the contactless vending machine, as well as strong appreciation for its impact on safety, dignity, and community well-being. Free-text comments provided by participants included the following statements:

- “Afraid I would get kicked out of shelter if someone saw me there.”
- “I live in [city in neighboring state approximately 2 h away from SSP], and it's hard to get a ride when its open.”
- “I wish there were satellite locations to pick up supplies outside of the city [in rural areas].”
- “I know a lot of people who have less scars and use new needles more often due to this service.”
- “I think the vending machine at [SSP] is a great help for a bad thing. Makes the sad reality of drug as safe as a user can possibly be. I wish more people knew about what they offer.”
- “I feel like most people don’t care if people like me live or die. This is one of the only places I’ve gone where people treat me like a human being and I appreciate that so much. I also want you to know that I’ve used Narcan from [the vending machine] to save someone’s life before.”

### Harm reduction self-efficacy

Participants reported the most confidence in their ability to “use a tourniquet to tie off rather than a belt or necktie” followed by their ability to “inject into arms or the back of legs before trying anywhere else,” to “do a test shot (use a smaller dose than usual) before injecting your drug,” to “use a clean cooker and clean cotton or filter when you inject,” to “get a brand new needle to inject,” to “use opioids without alcohol or other drugs in your system,” and to “do a taster shot before injecting your drug (let the tourniquet off after you insert the needle and before pushing in the plunger).” Participants reported the least confidence in their ability to “cut back on the amount of drug that you usually use,” to “use bleach and water to clean a used syringe before using it again,” and to “smoke or snort your drug if a vein is not available.” After correcting for multiple comparisons, there were no statistically significant differences by usual harm reduction supply method in harm reduction self-efficacy. Additional details regarding participants’ harm reduction self-efficacy are shown in Table 2.

**Table 2:** Harm reduction self-efficacy confidence scores of people who inject drugs by usual harm reduction supply access method (N = 50)

	Overall sample (*N* = 50) mean (SD)	In-person access (*n* = 12) mean (SD)	Contactless access (*n* = 38) mean (SD)	p-value
Cut back on the amount of drug that you usually use	3.9 (2.6)	4.5 (2.3)	3.7 (2.6)	0.360
Use opioids without alcohol or other drugs in your system	6.2 (3.7)	5.8 (4.3)	6.4 (3.5)	0.618
Do a test shot (use a smaller dose than usual) before injecting your drug	6.5 (3.0)	6.8 (3.6)	6.4 (2.9)	0.668
Do a taster shot before injecting your drug (let the tourniquet off after you insert the needle and before pushing in the plunger)	6.0 (3.3)	6.3 (3.7)	5.9 (3.2)	0.747
Use a clean cooker and clean cotton or filter when you inject	6.5 (3.2)	7.8 (2.6)	6.1 (3.3)	0.106
Take a warm bath, wear a sweater, or move your arms around to bring out a vein before trying to inject	5.6 (3.2)	5.2 (3.3)	5.7 (3.2)	0.594
Use a different injection site so old sites can heal	5.1 (3.0)	6.3 (3.0)	4.7 (2.9)	0.121
Wash and clean your injection sites before and after injecting	5.2 (2.7)	5.0 (2.9)	5.2 (2.7)	0.797
Inject into arms or the back of legs before trying anywhere else	6.8 (2.8)	7.2 (1.5)	6.7 (3.1)	0.645
Smoke or snort your drug if a vein is not available	4.6 (3.4)	4.7 (3.4)	4.5 (3.5)	0.904
Get a brand new needle to inject	6.5 (3.0)	7.2 (2.8)	6.3 (3.0)	0.381
Clean all surfaces where you will prepare your injection with soap and water	5.0 (3.3)	5.2 (3.6)	5.0 (3.3)	0.882
Use bleach and water to clean a used syringe before using it again	4.1 (3.3)	4.4 (3.5)	4.1 (3.3)	0.742
Choose a safe place to inject that is private, clean, well lit, and warm	5.3 (3.5)	7.1 (2.8)	4.8 (3.6)	0.045
Use a tourniquet to tie off rather than a belt or necktie	7.4 (2.9)	8.7 (1.9)	7.0 (3.0)	0.085
Total harm reduction self-efficacy score	84.8 (30.0)	91.9 (29.5)	82.6 (30.1)	0.352

## Discussion

In the context of the continued drug overdose crisis in the United States and Canada [4, 6, 7], and in the absence of impactful regulatory efforts to reduce the toxicity of the street drug supply, communities require innovative solutions to providing harm reduction services that are accessed by the people who need them. Traditional SSPs face barriers such as stigma, restrictive zoning laws, and limited operating hours, making it difficult for individuals to obtain sterile supplies and lifesaving medications like naloxone. The current pilot study demonstrates that some people who inject drugs choose to access harm reduction supplies via vending machine for various reasons, but most find this method to be more accessible and approachable than accessing in-person SSP services. Our exploratory findings also suggest that this preference may be driven out of acceptability and convenience rather than the attributes of people who inject drugs, given that we did not find any statistically significant differences in demographics by usual access method. However, it should be noted that the sample size was small, which limits our statistical power. It is possible that we may have been underpowered to detect small differences between these groups.

According to Bandura’s self-efficacy theory, when individuals perceive greater ease of access to essential supplies, their confidence in managing their own health behaviors is likely enhanced [1]. Participants using the vending machine reported greater convenience and autonomy, which may have positively impacted their sense of self-efficacy. However, the absence of direct interaction with staff may limit opportunities for education and personalized support, which are elements that could further strengthen self-efficacy in in-person settings. Of note, participants’ harm reduction self-efficacy related to using alternative routes of administration, such as smoking, was notably low compared to other assessed behaviors. This may be due, in part, to the fact that the SSP did not stock safer smoking supplies at the time of the survey, potentially limiting both awareness and confidence in these practices. As a result of these and other findings [24], the SSP has since added safer smoking supplies to its available inventory for both in-person and contactless access, aiming to better support non-injection routes of drug use. Smoking substances, when feasible, is generally considered less harmful than injection use, and has been associated with a lower risk of skin and soft tissue infection, hospitalization, and overdose [17].

Participant reflections revealed several intersecting themes that highlight both the promise and importance of contactless harm reduction services. Comments pointed to pervasive stigma and social marginalization, barriers related to geography and transportation, and the critical role of convenience in accessing supplies. Others emphasized the life-saving potential of these services, particularly through access to naloxone, as well as the value of anonymous, nonjudgmental engagement. Taken together, these themes align with broader harm reduction literature and reinforce the need for flexible, low-threshold models of care that meet people where they are, especially those who may not access traditional, in-person services. These insights have important implications for expanding harm reduction infrastructure and reducing disparities in access.

Research has shown that harm reduction vending machines effectively reduce syringe sharing, increase naloxone availability, and connect high-risk populations to supplies and services without contributing to an increase in drug use [12, 26]. Additionally, vending machines have been successfully implemented in various settings, demonstrating their potential as a cost-effective and scalable intervention. While critics argue that these vending machines may encourage drug use or pose public safety concerns, the overwhelming abundance of evidence suggests that they save lives and reduce the public health burden of injection drug use. In the current study, we found that there were no significant differences in the harm reduction self-efficacy of people who inject drugs based on the usual method of harm reduction supply access, suggesting that contactless methods of harm reduction supply access do not contribute to a reduced ability to engage in safer drug use practices. Given the continued rise in overdose deaths [15] and the failure of punitive drug policies in the United States [5], expanding contactless harm reduction supply methods like vending machines is a necessary and pragmatic step to reducing drug-related harms for people who inject drugs.

### Limitations

Our pilot study has some limitations. First, our sample size was small and not very racially or ethnically diverse. In general, disparities in access to harm reduction services remain, with marginalized communities often facing the most significant barriers [18]. The small sample size and unbalanced comparison groups limit our statistical power to detect significant differences between participants who accessed harm reduction supplies in person versus via vending machine. A post-hoc power analysis suggests that this study was powered to detect medium-to-large effect sizes. As such, smaller or more nuanced differences in harm reduction self-efficacy or participant characteristics may have gone undetected. Additionally, participants who reported usually accessing harm reduction supplies via vending machine may have also periodically sought in-person services where harm reduction education was delivered, thereby affecting their harm reduction self-efficacy. Participants for this study were recruited from a single SSP located in the state of Illinois, and our findings may not generalize to other populations of people who inject drugs. Regulations and public sentiment regarding SSPs vary widely across the United States [8]. While Illinois laws currently permit and actively support the implementation of SSPs with robust harm reduction services, these programs remain predominantly concentrated in urban areas [19]. Additionally, the broader sociopolitical climate must be considered; in recent years, there has been a growing national backlash against harm reduction efforts, including the distribution of naloxone and the operation of SSPs. This resistance may further limit the generalizability of our findings to regions where harm reduction services face legal, political, or cultural opposition. Our study was also exploratory in nature; these limitations underscore the need for larger and more representative studies to validate and expand upon these exploratory findings.. Additionally, qualitative research could supplement these findings by more richly examining the reasons why people who inject drugs access SSPs in different ways and how these access methods might affect the behaviors and self-efficacy of people who inject drugs. Subsequent research in partnership with the SSP examined in this study can explore these topics.

### Conclusions

Findings from the current pilot study suggest that although people who inject drugs may access SSPs in various ways, they do not meaningfully differ in terms of their demographics or harm reduction self-efficacy. Moreover, people who inject drugs find contactless harm reduction supply methods to be impactful and more convenient and approachable than in-person services. Taken together, our findings support reducing barriers to harm reduction services and expanding access to contactless harm reduction supply methods. When and where in-person syringe services cannot be provided, vending machines are an efficient method to maintain service delivery with reduced staffing requirements [7]. While contactless vending machines increase access to sterile supplies, especially in geographically remote or stigmatizing contexts, these services should complement, not replace, the critical role of in-person harm reduction programs, which provide vital interpersonal connection, education, and linkage to care. Addressing barriers to in-person SSP access remains essential to meeting the diverse needs of people who use drugs.

## Data Availability

Data will be provided upon reasonable request to the corresponding author.
